# Antimicrobial Stewardship in Pediatrics

**DOI:** 10.3390/antibiotics13010105

**Published:** 2024-01-22

**Authors:** Radoslaw Jaworski, Katarzyna Dzierzanowska-Fangrat

**Affiliations:** 1 Department of Pediatric Cardiology and Congenital Heart Defects, Medical University of Gdansk, 80-210 Gdansk, Poland; 2 Department of Clinical Microbiology and Immunology, Children’s Memorial Health Institute, 04-730 Warsaw, Poland; k.fangrat@ipczd.pl

Since the discovery of antibiotics in the early 20th century, significant changes have occurred in their usage principles. Initially, there was great optimism about this new class of medications. However, it became evident that the uncontrolled use of antibiotics in humans, veterinary medicine, and agriculture led to antimicrobial resistance (including the emergence of pandrug-resistant organisms), resulting in a global health emergency.

Currently, antimicrobial resistance (AMR) is a worldwide problem, contributing to millions of deaths each year [1]. In response to this crisis, the World Health Organization (WHO) adopted a global action plan for AMR in 2015, emphasizing the need for multisectoral collaboration and encouraging countries to develop their own national action plans [2]. In 2017, the European Commission launched EU Guidelines for the prudent use of antimicrobials in humans [3].

Currently, antimicrobial stewardship (AMS), defined as an organizational or healthcare-system-wide approach to promote and monitor the judicious use of antimicrobials to preserve their effectiveness, is emerging as a pivotal strategy in clinical settings [4]. All clinical settings are advised to implement AMS programs into their practice.

While much research has been published in this area, there are still many obstacles to implementing AMS into daily routines, especially in the pediatric population [5,6,7]. This Special Issue specifically encourages the submission of papers on antibiotic stewardship in pediatric settings. Submissions regarding the optimal use of antibiotics in general pediatrics, perioperative management in all surgical areas, pediatric hematology and oncology, and patients with primary immunodeficiency, among others, have been invited.

This Special Issue includes six articles: three original papers, one review, and two recommendations. The first paper (Amari et al., contribution 1) addresses the antibiotic resistance of Streptococcus pneumoniae isolated from healthy children under five years old, which began 8–10 years after the introduction of pneumococcal vaccination in Morocco. The authors found that the rate of multidrug resistance (17%) in S. pneumoniae was much lower than in many other African and Asian countries, suggesting that vaccination has an important role in AMR control. The second paper (Guarch-Ibanez et al., contribution 2) discusses the current status of pediatric antimicrobial stewardship programs in hospitals in Catalonia, Spain, and defines the necessary steps to optimize their implementation. The third paper (Burzynska et al., contribution 3) focuses on perioperative antibiotic prophylaxis (PAP) in pediatric cardiac surgery in Poland by comparing two regimens used for children undergoing elective cardiac surgery. The authors proved that a simplified regimen combined with restricted access to a prophylactic antibiotic (cefazoline) was safe and resulted in better PAP adherence and a reduction in postoperative antibiotic use. The fourth paper (Autore et al., contribution 4) presents the current recommendations for the management of pediatric urinary tract infections. Using the Delphi method, the authors developed a consensus document, based on updated works in the literature, including 21 statements referring to various aspects of UTIs in children. In addition, this Special Issue includes one review by Bossu et al. (contribution 5), which explores the problem of fungal infection prophylaxis and treatment in children with cancer. The authors discuss the latest update of the ECIL guidelines and provide insights into novel drugs and immunotherapy options in this patient group. Finally, the last article, submitted by a group from Italy, focuses on antibiotic prophylaxis for the prevention of urinary tract infections in children, and it contains 15 recommendations based on a systematic review of the current literature (contribution 6). The authors conclude that low-dose continuous antibiotic prophylaxis plays a limited role in preventing UTI recurrences in children and does not mitigate complications. Therefore, this method should be limited to selected groups of patients.

This Special Issue consolidates multidisciplinary investigations on antimicrobial stewardship in pediatrics. Given the overarching nature of this predicament, a comprehensive approach becomes imperative, aligning with the WHO’s global initiative to enhance proper antibiotic usage. This is necessary to avert an impending post-antibiotic era, wherein common infections may once again pose life-threatening risks.
